# Transtracheal Esophageal Stent Removal: A Case-Series

**DOI:** 10.4021/jocmr1216e

**Published:** 2013-02-25

**Authors:** Guillaume Buiret, Michel Guiraud, Jerome Pierron, Mathieu Schoeffler, Serge Duperret, Jacques Baulieux, Lionel Wander, Marc Poupart, Jean-Christian Pignat

**Affiliations:** a ENT and Cervicofaciale Surgery Unit, Croix-Rousse Hospital, Hospices Civils de Lyon, France; b ENT and Cervicofaciale Surgery Unit, Valence Hospital, France; c Anesthesiology Unit, Croix-Rousse Hospital, Hospices Civils de Lyon, France; d Intensive Care Unit, Croix-Rousse Hospital, Hospices Civils de Lyon, France; e Abdominal Surgery Unit, Croix-Rousse Hospital, Hospices Civils de Lyon, France; f Hepato-gastro-enterology Unit, Croix-Rousse Hospital, Hospices Civils de Lyon, France

**Keywords:** Benign esorespiratory fistula, Tracheal stent, Esophageal stent, Esophageal exclusion

## Abstract

Benign esophagorespiratory fistula is a rare but often lethal affection and difficult to cure. Possible treatments are surgery or esophageal stenting but may fail and cause respiratory failure. Two patients with spontaneous esophagorespiratory fistula after chemoradiotherapy for an esophageal malignancy were both treated by esophageal exclusion but esophageal stent were left in place. The esophageal stents were transtracheally removed through the fistula. The removals were successful, patients could leave Intensive Care Unit and returned home. Transtracheal esophageal stent removal is technically possible but very risky. Such situations must be avoided: esophageal stents must absolutely be removed before esophageal exclusion.

## Introduction

Benign Esophago Respiratory Fistula (BERF) is potentially lethal and difficult to cure. If possible a curative surgery should be considered [1]. The most common alternative is the exclusion by an Esophageal Stent (ES) [2]. A Tracheal Stent (TS) can also be added. Unfortunately in spontaneous BERF after Chemo Radio Therapy (CRT) of an esophageal malignancy, healing wound abilities are almost null. Failure of BERF sealing by ES occurs in 0 to 33% and is always lethal for the patients [3, 4]. No specific data concerning post-CRT BERF is available. After endoscopic treatment failure an option is to surgically exclude the esophagus for the esophageal posterior wall to make a new tracheal wall [5].

Two patients were referred to our university hospital multidisciplinary team. Both suffered from a post-CRT inoperable BERF, the esophagus been previously excluded after endoscopic treatment failure but ESs remained in place and caused respiratory failure. ESs were successfully transtracheally removed.

## Case Report

**Figure 1:**
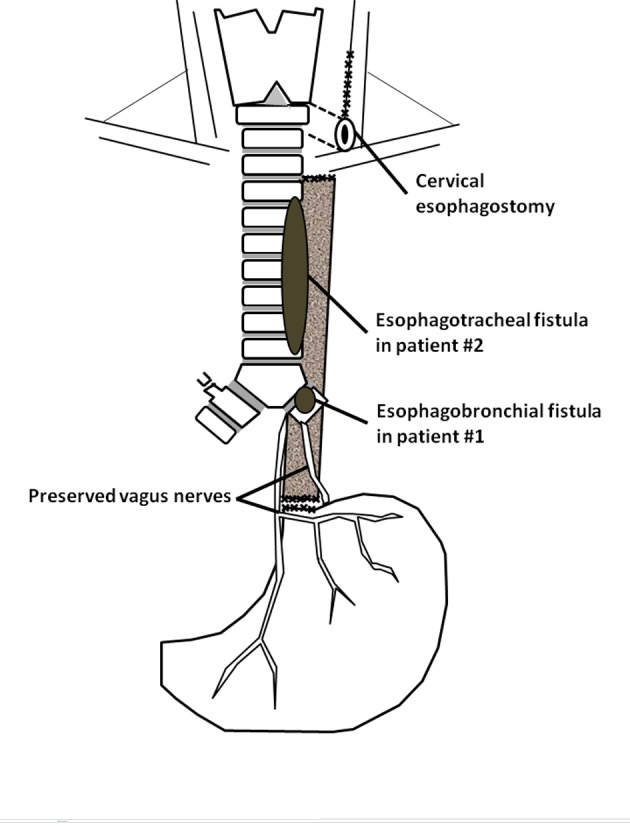
Surgical technique of bipolar esophagus exclusion (modified from Maillard et al [5]).

**Figure 2:**
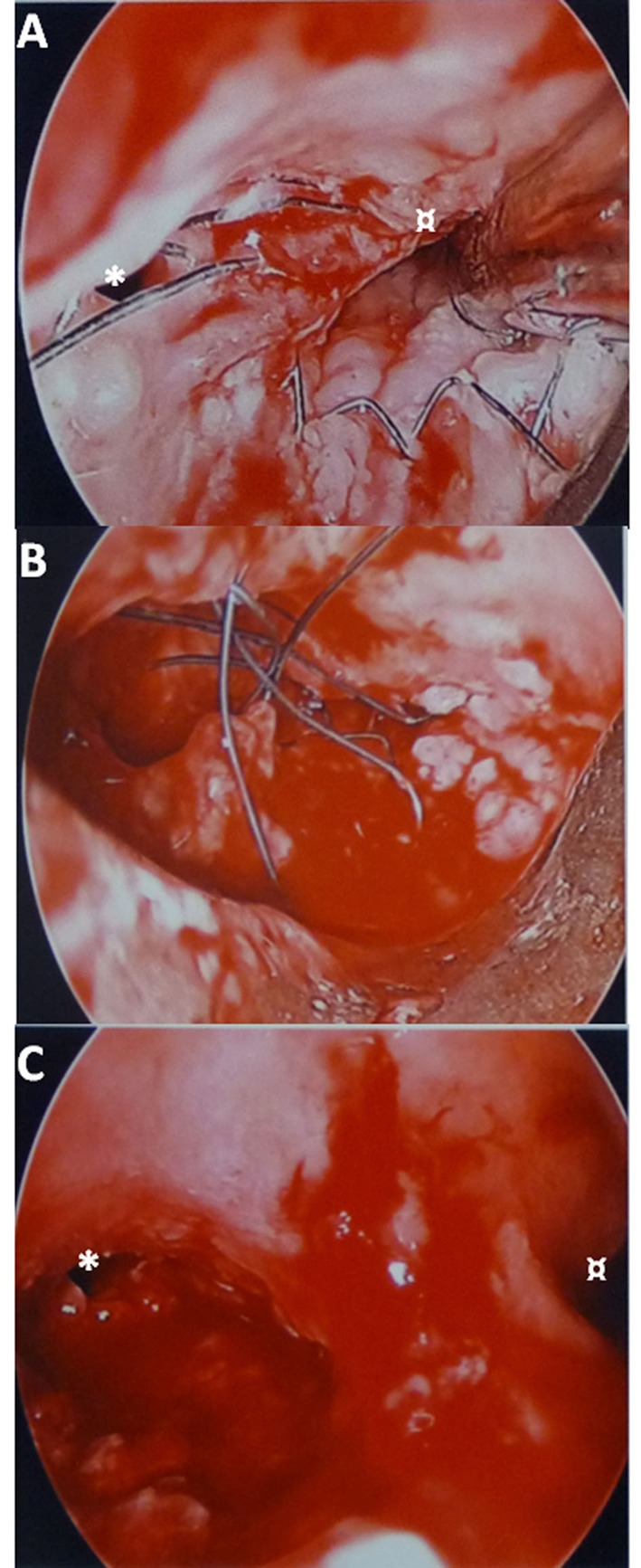
Endoscopic views of the main left bronchus in patient #1. A). Esophageal braiding in the main left bronchus. B). During stent removal, braid after braid. C). After removal. * Left upper lobar orifice. ¤ Left lower lobar orifice.

The patient #1 was a 62-year-old man referred to our Intensive Care Unit (ICU) to undergo a TS placement. His main past history included a T3N1 squamous cell carcinoma of the middle third of the esophagus treated by CRT in July 2010. He had just benefited from three ES placements in another hospital for a spontaneous BERF but this procedure failed and he was referred to our hospital. A rigid bronchoscopy was performed under General Anesthesia (GA) maintained with target controlled infusion of remifentanil and propofol without myorelaxant agent on March 24, 2011. A 2.5 cm-long defect of the posterior wall of the left main bronchus until the left lobar division did not allow a TS placement. Two of the three ESs, both totally covered Boston Wallflex® stents (Boston Scientific Inc, Watertown, MA, USA), were removed. The last ES, an uncovered Boston Ultraflex® stent (Boston Scientific Inc, Watertown, MA, USA), was completely covered by the esophageal mucosa and was not visible. It was not looked after under the mucosa to avoid any extension of the BERF. On March 25, 2011, the patient benefited from an esophageal exclusion with esophagogastric exclusion, retrosternal esophagogastroplasty and jejunostomy (Fig. 1). The patient was extubated on postoperative Day 3. The patient had purulent sputum and thus underwent many fiberscopic suctions: the braiding of the last ES was seen in the left main bronchus. On April 15, 2011, the last ES was removed under GA (Fig. 2). Breathing, spontaneous or controlled, was allowed through the endoscopic rigid tube, with monitoring of cardiac frequency, SpO2 and arterial pressure. Two erbium laser shots on the braiding divided it and the ES was removed braid by braid. Oral feeding was possible on Day 10, enteral feeding was stopped on Day 22. The patient left the ICU on Day 24 and hospital at Day 34. He was seen six months later without respiratory or swallowing complication.

**Figure 3:**
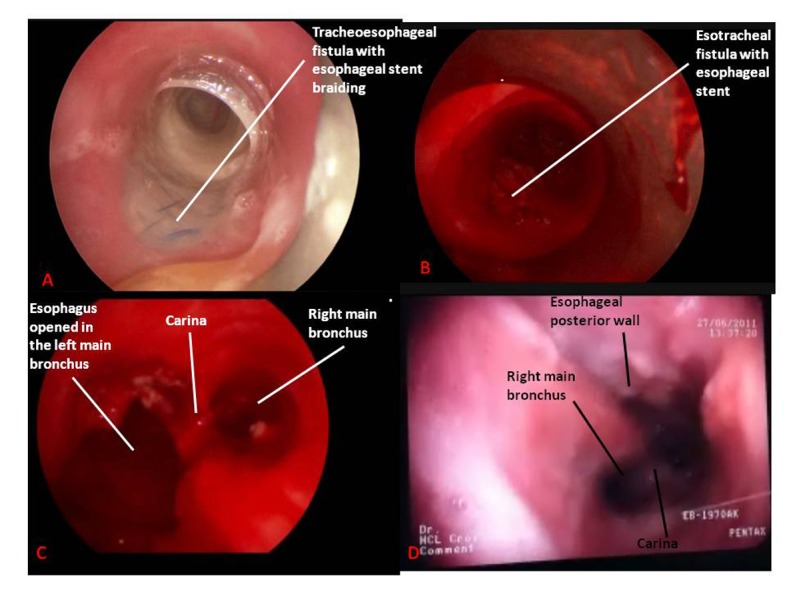
Endoscopic views in patient #2. A). Tracheal endoscopic view of tracheoesophageal fistula before stents removal. B). Tracheal endoscopic view of tracheoesophageal fistula after tracheal stent removal and before esophageal stents removal. C). Tracheal endoscopic view of tracheoesophageal fistula after tracheal stent and esophageal stents removal. D). Tracheal fiberoptic view three weeks after removal procedure.

The patient #2 was a 49-year-old woman transferred intubated in our ICU to undergo a TS placement. Her main past history was included a T3N1 squamous cell carcinoma of the middle third of the esophagus treated by CRT on June 2010. She had benefited from the placement of three totally covered Boston Wallflex® stents (Boston Scientific Inc, Watertown, MA, USA) some days earlier for a spontaneous BERF. Aspiration pneumonia provoked a respiratory failure and she was intubated. A visceral surgeon performed an esophageal exclusion with jejunostomy and cervical esophagostomy (Fig. 1) to decrease aspiration but left ESs inside the esophagus. On April 15, 2011, she benefited from a rigid bronchoscopy under GA (same protocol than patient #1): a 1 cm-diameter non-vital zone of the posterior tracheal wall 2 cm above the carina was seen. This was followed by 2 cm of normal mucosa then a 3 cm-long defect through which an ES was seen, finishing 5 cm under the vocal cords. A Novatech Y® (Novatech SA, La Ciotat, France) 16-13-13 was placed and the patient returned intubated to ICU. As the patient accidentally auto-extubated 3 times a tracheotomy was performed on May 6, 2011, with the lower end of the tube being placed into the TS. Afterwards the patient was ventilated with Pressure-Support Ventilation (PSV) during the day and Bilevel Positive Airway Pressure at night and finally with PSV during night and day. As the patient suffered from a multiresistant Pseudomonas aeruginosa pneumonia and had a lot of purulent sputum, a new bronchoscopy under GA was performed on June 6, 2011. The defect enlarged beginning 5 cm under the vocal cords but continued to the lower end of the left main bronchus. Thoracic surgeons were asked about the open surgery possibilities of ES removal but it was judged too risky because of her past history of RCT, the hemorrhagic risk and her current poor pulmonary condition. The ES was transtracheally removed on June 10, 2011 under GA, with the patient being ventilated through the bronchoscopic tube or tracheotomy (Fig. 3). She went back to ICU in PSV. On June 12, 2011, she could be weaned off ventilation during 4 - 6 hr/d and rehabilitation was initiated. On July10, 2011, tracheotomy tube was removed and she left ICU on July 21, 2011, and returned home on August 16, 2011. She benefited from a retrosternal esophagogastroplasty on February 2, 2012.

## Discussion

Transtracheal esophageal stents removal was technically possible whether the prosthesis was covered or not. No other removal solution, especially opened surgery, was considered by the multidisciplinary team (gastroenterology endoscopist, ENT surgeons, thoracic and visceral surgeons, ICU care providers) but the most important consideration is to prevent such difficult situations by removing every ES before esophageal exclusion, even uncovered ES completely recovered by esophageal mucosa. The uncovered ES removal was less risky because it was removed braid after braid in a patient with a better pulmonary condition and in spontaneous ventilation. The O_2_ was continuously delivered through the rigid bronchoscope. The first patient was able to eat and breathe without ventilation supply shortly after surgeries and left the hospital after less than one month. In contrast, covered ES removal in the second patient was very risky in many levels: TS removal first with the risk of respiratory collapse, the risk of enlarging the ES and causing a mediastinitis, the necessity to quickly exchange O_2_ delivery (through rigid bronchoscope or tracheotomy). The removal was technically possible thanks to very favorable conditions (very large BERF and a tracheotomy). Other conditions would certainly have prevented success of such a procedure.

Many ES types exist but expandable ES, covered or not, are less dangerous to place than non-expandable ones [2], especially those containing nitinol [4]. In the management of BERF expandable covered ES are used but placement and post-placement phases are not danger-less [6] particularly in this context of post-CRT spontaneous BERF because complications seems more frequent [7] (increase risk of bleeding, esophageal perforation and death of the patient) even if some authors disagree, especially Raijmann et al [3, 8]. Even though ACG guidelines [2] concluded that the evidence for BERF treatment by ES was very low and the strength of recommendation was weak, esophageal stenting with or without tracheal stenting can be successful in fragile patients and allow them to return home with their fistula being sealed off, temporarily or definitely [3].

In case of failure of esophageal stenting, esophageal exclusion is an appropriate treatment but ES must absolutely be removed before surgery. If not, the pulmonary condition of the patient will be seriously impaired and stent removal will technically be very complicated whether with opened or endoscopic surgery.
